# Relationship between Some Environmental and Climatic Factors on Outbreak of Whiteflies, the Human Annoying Insects

**DOI:** 10.18502/jad.v14i1.2714

**Published:** 2020-03-31

**Authors:** Abedin Saghafipour, Alireza Zahraei-Ramazani, Hassan Vatandoost, Amin Asadollahi, Reza Fouladi-Fard, Amir Hamta, Ali Hasanwand

**Affiliations:** 1 Department of Public Health, Faculty of Health, Qom University of Medical Sciences, Qom, Iran; 2 Department of Medical Entomology and Vector Control, School of Public Health, Tehran University of Medical Sciences, Tehran, Iran; 3 Department of Environmental Chemical Pollutants and Pesticides, Institute for Environmental Research, Tehran University of Medical Sciences, Tehran, Iran; 4 Research Center for Environmental Pollutants, Qom University of Medical Sciences, Qom, Iran; 5 Clinical Research Development Center (CRDU), Qom University of Medical Sciences, Qom, Iran

**Keywords:** Change point analysis, Climatic factors, Environmental change, Outbreak, Tehran

## Abstract

**Background:**

The reports of numerous outbreaks of whiteflies from different parts of the world have increased its medical importance. The aim of this study was to determine relationship between environmental changes and climatic factors with the outbreak of the whitefly population in Tehran, the capital of Iran.

**Methods:**

This study was carried out in urban areas of Tehran, where the increasing population of whiteflies was reported frequently during 2018. In order to entrap the whiteflies, 20 yellow sticky cards smeared with white refined grease were installed on the trunks of the trees at twice per month as trapping time intervals. The captured flies were transferred and conserved in cans containing 70% alcohol and were counted accurately under a stereomicroscope. To determine the relationship between air quality index, precipitation, air temperature and air humidity as environmental and climatic factors with the abundance of whiteflies, change point analysis and Generalized Estimating Equations (GEE) was used.

**Results:**

The most density of white flies per trap was 256.6 and 155.6 in early October and late September respectively. The number moved closer to zero from November to April. The population of whiteflies was inversely correlated with the level of air quality index (p= 0.99) and precipitation (p= 0.95), and it had a direct correlation with the high temperature. Also, the population of whiteflies had a direct correlation with the level of air humidity in the first half of the year

**Conclusion:**

According to these findings, during spring and summer from early May to early October.

## Introduction

Whiteflies (Hemiptera: Aleyrodidae) feed on a wide range of hosts in a way that for some species more than 900 plant species have been identified (1, 2). Cucurbits and ornamental plants, agricultural crops, palms, and weeds are the main hosts of this pest, though there are many weeds which are the secondary hosts (3). The life cycle of whiteflies from egg to adult complete one month depending upon environmental temperature. Adult whiteflies may be surviving for one to two months (4). This insect is considered a health problem and an important medical pest that can also threaten human safety in some cases. Accidental entry of a whitefly into the human respiratory tract can cause inflammation and infection in the upper respiratory tract leading to the emergence of opportunistic fungal and bacterial infections (5). The population of this insect has increased in many parts of the world which has caused many problems for humans, especially in urban areas (6). According to experts, repeated and uncontrolled use of various formulations and concentrations of pesticides can have many adverse effects, such as the resistance of pests to pesticides, the emergence of new pests, and the eradication of natural enemies (parasitoids and predators). Whiteflies are among the pests that have been evolved by continuous use of chemicals and the lack of proper management of pesticides (7). In addition to direct physical and biological harm for human, these insects cause a sharp decline in the production of agricultural products. Also, these flies cause the growth and development of saprophytic fungi on their honeydew which reduces the quality and nutritional value, as well as the consistency and shape of the products. Moreover, they physically damage non-productive plants. Nowadays, whiteflies, due to their increased resistance to various types of pesticides (8), cause more damage to a large number of crops and ornamental plants. Male and female winged insects feed on the leaf juice of plants. This leads to yellow spots on the leaves that directly damage the host plant and make it seem short and sick. Insect secretions on plant leaves can cause fungal growth (9). So far, about 1556 species of whiteflies have been identified in different parts of the world (10). Whiteflies in Iran were identified for the first time during the faunistic research and the identification of flies in Fars Province in 1995 (11). After that 14, 18 and 24 species of whiteflies have been reported in Isfahan (9), Gilan (12) and Golestan Provinces respectively (13). Subsequently, in 2000, morphological and biological studies were conducted on common species in Esfahan, which revealed that whiteflies in Esfahan were of the European race (9). Such flies are greenhouse pests, but, unfortunately, the lack of proper management of chemicals and pesticides has caused resistance to some of the pesticides and adverse environmental conditions in these insects. As a result, these flies have been able to adapt themselves to greenhouse conditions and easily grow and reproduce. Some experts also believe that these flies have been released into the open air through whitefly infestations, and since the ecosystem cycle and the population of predator insects have not been balanced in the environment, this has led to a widespread outbreak of whiteflies in the open air (14). Moreover, since these insects are expected to reproduce in places more similar to greenhouse in terms of climate and food resources, it is necessary to identify the ecological factors in the reproduction of the insects, and take measures to control them outdoors (15). Over the past few years, the outbreak of whiteflies in Iran, especially in the residential areas of Tehran, has caused different allergies to humans. Therefore, the present ecological study was conducted with the aim of determining the relationship between some environmental and climatic factors on outbreak of whiteflies, the human annoying insects Tehran; District 6 in 2018.

## Materials and Methods

### Study area

**Fig. 1.**
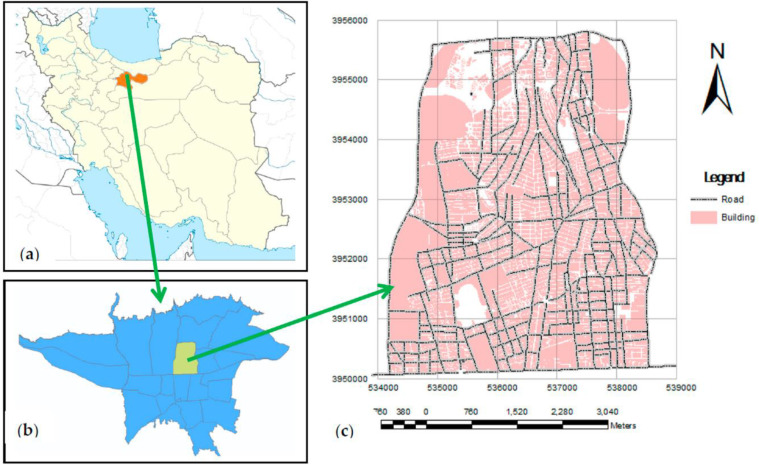
Geographic location of the study area in District 6, Municipality of Tehran, Iran

The current study was carried out in the urban area of Tehran; the capital of Iran (District 6) suffers from a severe air quality index, where the abundance of whiteflies has been reported frequently during 2018 (Fig. 1).

The city of Tehran is located between the mountainous region and the plain. Tehran’s climate is generally described as mild in spring and fall, hot and dry in summer, and cold and wet in winter. Based on the 2016 census, Tehran has a population of approximately 10 million. Vegetation coverage in Tehran is including natural and hand-planted forests (16). The air pollution indicators including Air Quality Index (AQI) with monthly activity of whiteflies were received from the Iran Meteorological Organization (17).

### Study design

To entrap whiteflies, 20 yellow sticky cards (fly traps) of 30×50cm (18), depending on the diameter of the tree, were installed on the trees each time at twice per month as trapping time intervals from Early April to late March 2018. Totally 480 yellow sticky cards have been applied for catching adult whiteflies. In addition, we replaced the old traps with new one in each visit.

The chemicals on the traps were odorless and smeared with purified white grease purchased wholesale from reputable stores. The traps were installed on the trees of Maple, Acacia, European ash, Sycamore, and Berries for the purpose of catching whiteflies. In this regard, to examine the effect of colorful traps on whiteflies, three different colors of yellow, blue and green for attracting and capturing of them were used in a selected greenhouse. In this study, colored cards of 10×22cm in two heights of 1.3 and 2.3m were installed on tomato plants. After two weeks, the numbers of trapped insects on colored cards were counted.

Whiteflies were isolated and counted in two ways: traps (sticky cards) were immersed in warm water for 2 minutes (min) until the grease on the surface containing the flies was softened, and the flies were released in hot water. Then, the insects were removed from the hot water using a brush and were transferred and conserved in cans containing 70% alcohol. Then, at appropriate times, the whiteflies in the canned glass were released into the appropriate plates and carefully counted under a stereomicroscope. In another method, the traps containing whiteflies were peeled off the trees, and given the grid pattern on the surface of traps, the number of flies in every grid was randomly counted and the total number of whiteflies was estimated on the surface of the traps. The yellow and blue colored traps used in this study were supplied by Russel IPM Company; and the green traps were made of Tangle foot adhesive supplied by Kerman Chemistry Company (applied on green cards with brush).

### Statistical analysis

Change analysis is organized to answer two questions 1) whether there are any change points or not 2) if so in which times change point(s) occurs. We deal with Hypothesis test and estimation in first and second question, respectively.

Generally, suppose *X_1_*, *X_2_*, …, *X_n_* to be sequence of independent random variables with probability distribution function *F_1_*, *F_2_*, …, *F_n_* respectively. Change point analysis intended to test following null hypothesis H_0:_ *F_1_= F_2_=* … *= F_n_* versus alternative hypothesis H_1:_ *F_1_=*⋯ F_k_1__≠*F*_*k*_1_+1_⋯=*F*_*k*_2__≠*F*_*k*_1_+1_=*F*_*k*_*q*__≠*F*_*k*_*q*_+1_=⋯=*F_n_*

Where 1< *K_1_*< *K_2_*< ⋯ < *K _q_*< *n* and *q* is unknown parameter that shows number of change points and *K_1_*, *K_2_*, ⋯, *X_q_* are the change points and have to be estimated. Although in majority of studies which point analysis has been applied probability distribution function supposed to be normal (19), in current study with the respect to response variable, it is let be Poisson. So our problem to detect change point (s) refor to *X_1_*, *X_2_*, ⋯, *X_c_* that are consequence of Poisson random variable with parameters λ_i_, i_=_ 1, 2, c respectively. The aim was to test following null hypothesis H_0:_ *λ_1_=* ⋯*λ_c_* versus alternative hypothesis

$$H_{1}:\lambda_{1}=\cdots =\lambda_{k}=\lambda \neq \lambda_{k+1}\ldots =\lambda_{c}=\lambda^{\prime }$$

that shows there are k unknown change points. Likelihood function based on null hypothesis is:

$$L_{0}(\lambda )=\frac{e^{-c\lambda }\lambda^{\sum_{i=1}^{c}x_{i}}}{\prod_{i=1}^{c}x_{i}!}$$

so likelihood estimation based on that is:

$$\hat{\lambda }=\frac{\sum_{i=1}^{c}x_{i}}{c}$$

and under alternative hypothesis likelihood function is obtained

$$L_{1}\left(\lambda .\lambda^{\prime }\right)=\frac{e^{-k\lambda }\lambda^{\sum_{i=1}^{k}x_{i}}}{\prod_{i=1}^{k}x_{i}!}.\frac{e^{-(c-k)\lambda^{\prime }}{\lambda^{\prime }}^{\sum_{i=k+1}^{c}x_{i}}}{\prod_{i=k+1}^{c}x_{i}!}$$

by letting

$$\begin{array}{l} M_{k}=\sum_{i=1}^{k}x_{i},\\ M_{k}{}^{\prime }=M-M_{k}=\sum_{i=k+1}^{c}x_{i} \end{array}M=\sum_{i=1}^{c}x_{i},$$

the likelihood estimation function of *λ* and *λ* ^′^ are given by $\hat{\lambda }=\frac{M_{k}}{c},{\hat{\lambda }}^{\prime }=\frac{{M^{\prime }}_{k}}{c-k}$ .

SIC ^1^ under null hypothesis is found as

$$\mathrm{SIC}(c)=-2\log L_{0}\left(\hat{\lambda }\right)+\mathrm{logc}$$

and corresponding alternative hypothesis determined from

$$\mathrm{SIC}(k)=-2\log L_{1}\left(\hat{\lambda }.{\hat{\lambda }}^{\prime }\right)+2\mathrm{logc}$$

So, according to the information criterion principle *H*_ 0_ was rejected if $\mathrm{SIC}(c)>\underset{1<k<c-1}{\text{min}}\mathrm{SIC}(k)$ . In order to estimating change point(s) $\mathrm{SIC}(\hat{k})>\underset{1<k<c-1}{\text{min}}\mathrm{SIC}(k)$ was used (19, 20). After determination of change point (s) in the next stage for considering correlation between data, which were collected over time, marginal longitudinal model was applied. Link function because of being count response variable, log Poisson fulfilled. Coefficients were estimated by Generalized Estimating Equations (GEE). This method provides predictive model for the response variable by explanatory variables and it takes into account possible correlation between repeated measures of depend variable of a subject. In this study since data are collected over time it is likely that repeated measure on an individuals are correlated. When GEE method is used in order to analyze longitudinal data, correlation structure is formulated and possible correlations between measurements over time is incorporated (21).

Suppose that *Y*_*ij*_ is a count and we are interested to investigate changes in expected count to the covariates. Since counts are often modeled as poisson random variable, using poisson variance function and log link function, marginal model for *Y*_*ij*_ would be illustrated as follow:

The mean of *Y*_*ij*_ (*μ*_*ij*_) is related to the independent variables by log link function:
  $$\text{log}\left(\mu_{ij}\right)=X_{ij}^{'}\beta$$
Also the variance of each *Y*_*ij*_ depends on *μ*_*ij*_:
  $$\mathrm{Var}\left(Y_{ij}\right)=\phi \mu_{ij}$$
In addition to these, an unstructured pairwise correlation pattern is assumed for the within-subject association among the repeated responses vector:
  $$\mathrm{corr}\left(Y_{ij},Y_{ik}\right)=\alpha_{jk}$$

The vector of parameters *α* shows the pairwise correlation among responses.

The marginal model specified above is a log-linear regression model, with an extra-poisson variance assumption (22).

So, to determine the relationship between air quality index, precipitation, air temperature and air humidity as environmental and climatic factors with the abundance of whiteflies this model was applied. The goodness of fit of model was evaluated by QIC.

## Results

**Fig. 2.**
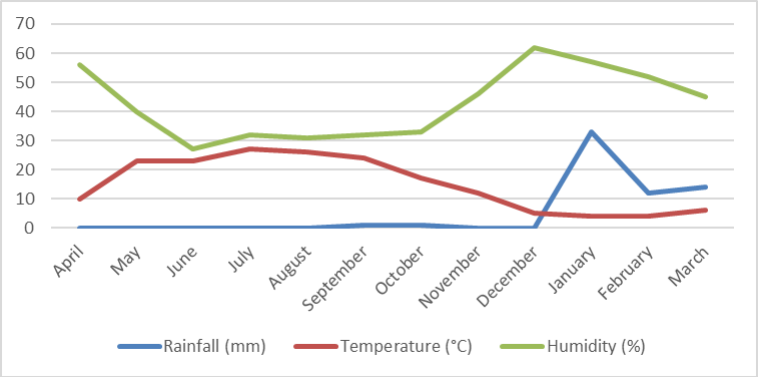
Average monthly rainfall, temperature and humidity, Tehran, Iran in 2018

The density of whiteflies per trap in different seasons were calculated during the 12-month period of sampling in district No. 6 of Tehran in 2018. The most density of white flies per trap was 256.6 and 155.6 in early October and late September respectively due to low temperature and rainfall and high humidity (Fig. 2). The density of whiteflies per trap in other times of year was shown in Table 1. The number moved closer to zero from November to April. The population of whiteflies was inversely correlated with the level of air quality index (p= 0.99) and precipitation (p= 0.95), and it had a direct correlation with the high temperature. Also, the population of whiteflies had a direct correlation with the level of air humidity in the first half of the year, and it was inversely correlated in the final months of the year.

**Fig. 3.**
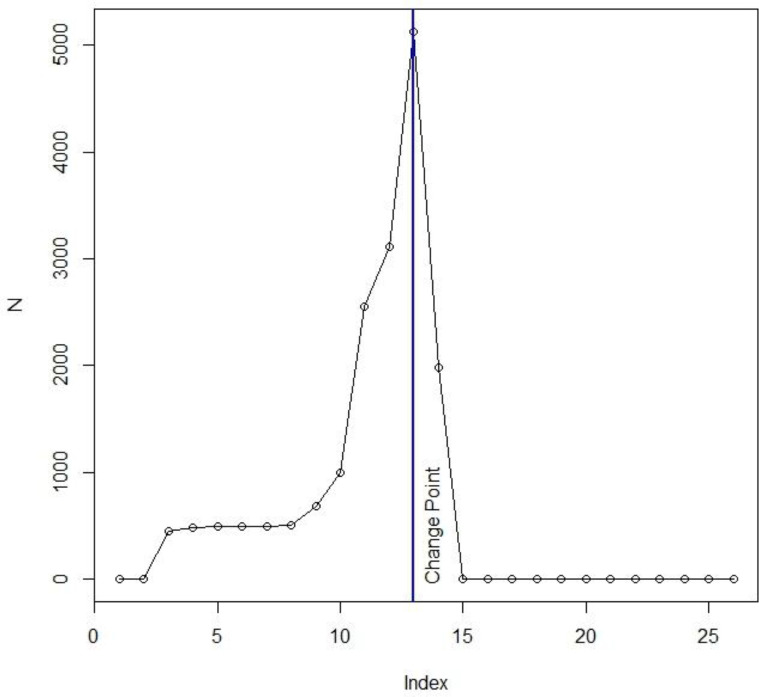
Change point of whiteflies activity, Tehran, Iran, 2018 (N= number of collected whiteflies; Index= times of trapping)

**Table 1.** Comparison of Air Quality Index (AQI) with monthly activity of whiteflies, Tehran, Iran in 2018

**Variable**	**Density of whitefly per trap**	**Air quality index (AQI)**

**Month**		
**Early April**	00.00	71.52
**Late April**	00.00	71.68
**Early May**	22.55	75.53
**Late May**	24.45	79.56
**Early June**	24.70	82.93
**Late June**	24.75	94.31
**Early July**	24.55	93.67
**Late July**	25.45	87.81
**Early August**	34.00	89.47
**Late August**	50.00	90.75
**Early September**	127.8	102.73
**Late September**	155.6	100.68
**Early October**	256.6	88.00
**Late October**	99.25	84.47
**Early November**	00.00	93.06
**Late November**	00.00	128.12
**Early December**	00.00	136.15
**Late December**	00.00	138.14
**Early January**	00.00	142.28
**Late January**	00.00	145.85
**Early February**	00.00	121.45
**Late February**	00.00	112.15
**Early March**	00.00	110.59
**Late March**	00.00	108.14

In the current study, up to the 13^th^ whiteflies trapping (early October), using the marginal model, the estimates were calculated by the GEE method, and considering the Poisson link function, the following results were obtained:

- For a unit of increase in air quality index (AQI), the population of whiteflies decreased by 0.98 (decreases).
- For a unit of increase in temperature, the population of whiteflies increased by 1.18 (increases).
- For a unit of increase in humidity, the population of whiteflies increased by 1.04 (increases).
- For a unit of increase in precipitation, the population of whiteflies decreased by 0.99 (decreases) (Table 2). In this method, all four variables were significant.

In addition, the following results were obtained after the 13^th^ measurement (early October):

- For a unit of increase in air quality index (AQI), the population of whiteflies decreased by 0.98 (decreases).
- For a unit of increase in temperature, the population of whiteflies increased by 2.37 (increases).
- For a unit of increase in humidity, the population of whiteflies decreased by 0.92 (decreases).
- For a unit of increase in precipitation, the population of whiteflies decreased by 0.44 (decreases) (Table 3). In this method, none of the four variables were significant. In both GEE models, QIC showed models were well-fitted.

**Table 2.** Generalized estimating equation (GEE) models predicting number of flies before 13th measurement, Tehran, Iran, 2018

**Variable**	**Coefficient**	**S.E.**	**P- value**
**Air pollution**	0.98	0.001	<0.001
**Temperature**	1.18	0.004	<0.001
**Humidity**	1.04	0.003	<0.001
**Rain fall**	0.99	0.000	<0.001
**Constant**	12.98	2.65	<0.001

## Discussion

The results of this study showed that the population of the whiteflies was inversely correlated with the level of air quality index and precipitation, and it had a direct correlation with high temperature. Also, the population of whiteflies had a direct correlation with the level of air humidity in the first half of the year, and it was inversely correlated in the final months of the year.

**Table 3.** Generalized estimating equation (GEE) models predicting number of flies after 13th whiteflies trapping, Tehran, Iran in 2018

**Variable**	**Coefficient**	**S.E.**	**P- value**
**Air pollution**	0.98	7.40	0.99
**Temperature**	2.37	3.31	0.53
**Humidity**	0.92	<0.0001	-
**Rain fall**	0.44	6.35	0.95
**Constant**	1	556.34	1.00

Modern transportation and rapid trading of plants, cuttings, branches and other parts of plants, which often contain eggs, larvae and whitefly nymph, have led to the transfer of these pests into new places. However, there may be few whiteflies that enter a new place independently and without encountering the natural enemies with which they are evolving. Appropriate level of humidity and temperature is another factor for the developing population of whiteflies. The whiteflies were commonly able to complete their life cycle including egg, larvae, nymph and adult in the temperature of 15–35 °C while survival was usually reduced at temperatures <20 °C or >30 °C (23, 24). The rate of survival of the whiteflies at unfavorable low and high temperatures were also affected by host plants (25). For instance, according to life span of mulberry trees in Tehran City, white flies strongly infest these trees (26) and when the leaves fall, the whiteflies population also decreases.

Importing of seedlings and cuttings of flowers, as well as the types of wood susceptible to the growth of whiteflies are one of the most important factors in the spread of whiteflies in different places. Whiteflies unable to fly long distances (27). Also, lack of precipitation is another factor in the survival of whiteflies. The high level of pollutants containing CO_2_ in the air of Tehran, due to defective fuel consumption of worn out vehicles and other airborne pollution, is another important factor in the survival of whiteflies in urban areas since these flies live in conditions similar to greenhouse. It should be mentioned that, in fact, the high level of pollution and weather conditions are important factors in the reproduction of whiteflies in Tehran (28). The abundance of whiteflies in Tehran can cause health problems including itching, red and sore eyes, runny nose, allergies, and problems in the respiratory system of individuals, especially asthmatics (29). Children and people with poor immune system, the elderly populations and pregnant women are more susceptible to these problems. Also, the presence of whiteflies in food and beverages, besides causing fear, is concerning in terms of contamination with pathogenic microorganisms. Therefore, this study was conducted in different months of the year to consider the effects of temperature, precipitation, humidity and environmental contaminants. The materials on the wings and bodies of the whiteflies can act as the pollen and cause allergic reactions in individuals (30). Rain and low humidity (below 60%) and low or high temperatures disrupt development of the insects. Whiteflies stay in one place because they cannot migrate long distances. The longest reported displacement of this insect is 7km (31, 32). Whiteflies can also cause problems for the eyes and respiratory tracts (31). The reasons for eradication of the natural enemies of whiteflies include the destruction of their reproductive habitats, uncontrolled use of authorized and unauthorized pesticides, and the lack of using specialized biological controlling methods. These factors influence the growth of whiteflies in metropolitan cities like Tehran. Reducing the use of smoky and pollutant vehicles and monitoring the factories that produce vehicles with incomplete combustion (33, 34) is one of the major factors in making the environment unstable for whiteflies. The use of different least-hazardous pesticides to reduce the resistance of whiteflies, as well as the use of systemic toxins for non-productive trees (35), and reducing the use of insecticides as the only methods of controlling whiteflies, and ultimately the use of growth regulators and integrated pest management (36) will help control these pests in such cities as Tehran.

## Conclusion

It should be mentioned that, in fact, the high level of pollution in different times of year and weather conditions are important factors in the reproduction of whiteflies in Tehran. According to these findings, during spring and summer from early May to early October that temperature and humidity for development of withe flies are supplied in Tehran City, personal protection against these pests was recommended by Tehran residents.

## Footnotes

1 Schwarz information criterion
